# Case Report: Aquaporin‐4–Positive Neuromyelitis Optica Spectrum Disorder: Diagnostic Challenges and Transition From B‐Cell Depletion Therapy to Ravulizumab

**DOI:** 10.1002/ccr3.73704

**Published:** 2026-10-12

**Authors:** Aram Zabeti

**Affiliations:** ^1^ Department of Neurology and Rehabilitation Medicine, Waddell Center for Multiple Sclerosis University of Cincinnati Cincinnati Ohio USA

**Keywords:** aquaporins, complement C5, diagnosis, neuromyelitis optica spectrum disorder, ravulizumab, rituximab

## Abstract

This case report illustrates the real‐world efficacy of complement component 5 inhibitors in a patient with neuromyelitis optica spectrum disorder who switched from rituximab because of B‐cell repopulation occurring early in the course of treatment. Maintenance of remission without relapse was observed when transitioning from rituximab to eculizumab and then to ravulizumab. Results observed in this case support the use of targeted complement inhibition as relapse‐preventive therapy after rituximab.

## Key Clinical Message

This case report describes the real‐world scenario of a patient with neuromyelitis optica spectrum disorders transitioning from B‐cell depletion (rituximab) to complement 5 inhibition (eculizumab then ravulizumab). This case highlights the efficacy of this approach and the importance of early diagnosis and treatment to prevent or reduce relapses and disability.

## 1 Introduction

Neuromyelitis optica spectrum disorder (NMOSD) comprises rare autoimmune inflammatory disorders of the central nervous system characterized by recurrent optic neuritis and longitudinally extensive transverse myelitis [1, 2, 3]. The disorders occur in approximately 0.5–10 people per 100,000 globally and predominantly affect women (female/male ratio, 3:1–10:1) [2, 3]. In patients with NMOSD, recurrent, unpredictable attacks of the optic nerves, spinal cord, and other regions of the central nervous system often lead to severe neurological disability, including visual impairment, motor dysfunction, sensory disturbances, autonomic bladder or bowel dysfunction, neuropathic pain, or area postrema syndrome (bouts of intractable nausea, vomiting, and/or hiccups), severely affecting the patient's quality of life [2].

In most cases, NMOSD is associated with pathogenic immunoglobulin G (IgG) antibodies against aquaporin 4 (AQP4), a water channel protein highly expressed in astrocytes in the central nervous system [1, 4]. Binding of AQP4‐IgG antibodies to AQP4 contributes to the immunopathogenesis of NMOSD by activating the classical complement pathway. The resulting cleavage of the terminal complement component 5 (C5) into C5a and C5b leads to inflammation, formation of the membrane attack complex, and subsequent astrocyte destruction and neuronal injury [5, 6, 7].

Given the high risk for relapse and accumulating neurological damage, long‐term immunotherapy, initiated as soon as a diagnosis of AQP4‐Ab+ NMOSD is established, is recommended for preventing attacks or reducing their frequency and severity [8]. Rituximab, a monoclonal antibody targeting CD20+ B cells, is a widely used therapy (mostly off‐label) for relapse prevention, but some patients receiving rituximab continue to have recurrent attacks, experience breakthrough attacks, and/or develop hypogammaglobulinemia, systemic infections, or other adverse effects [8, 9, 10, 11]. Multiple targeted monoclonal antibodies are approved for the treatment of AQP4‐Ab+ NMOSD to maintain disease remission, including inebilizumab, which depletes CD19+ B cells, satralizumab, which blocks the interleukin‐6 pathway, and eculizumab and ravulizumab, which inhibit terminal complement C5 [8, 11].

Considering the critical role of AQP4 in the pathogenesis of NMOSD [1], the development of C5 inhibitors was an important advance in their treatment. Both eculizumab and ravulizumab are humanized monoclonal antibodies that specifically bind and inhibit C5, preventing its cleavage into C5a and C5b and subsequent initiation of the membrane attack complex, which causes severe damage to astrocytes and subsequent inflammation [12, 13]. Ravulizumab is a second‐generation C5 inhibitor based on eculizumab, but it has a longer half‐life and extended dosing interval (intravenously once every 8 weeks [Q8W] versus 2 weeks [Q2W] for eculizumab) [13]. In the pivotal Phase 3 trials, eculizumab led to a 94.2% reduction in NMOSD relapse risk and ravulizumab to a 98.6% reduction in relapse risk versus placebo in patients with AQP4‐Ab+ NMOSD [12, 13].

Limited real‐world data exist on use of the recently approved long‐lasting C5 inhibitor ravulizumab [8, 13], particularly in patients who have transitioned from other treatments such as B‐cell depletion therapy and/or eculizumab. This case report describes the real‐world clinical scenario of a patient transitioning from B‐cell depletion therapy to the C5 inhibitors eculizumab and then ravulizumab and discusses practical considerations such as the timing of transition, vaccination requirements, and logistics. In addition, this report details diagnostic challenges associated with NMOSD; the patient's atypical gastrointestinal symptoms at presentation and a limited awareness of NMOSD among non‐neurologists may have contributed to a substantial delay in obtaining a neurological physical examination, magnetic resonance imaging (MRI), and serum AQP4‐IgG testing, none of which were performed until the patient developed acute and more obvious neurological symptoms and physical disability. This complex case report highlights the urgency for disease education and early diagnosis to minimize relapses and disability in patients with NMOSD.

## 2 Case History/Examination

A 29‐year‐old African American female with a medical history of allergy, asthma, and upper respiratory infection initially presented to an emergency department with a viral respiratory infection followed by pleurisy. In the 4 months after the initial presentation, the patient had multiple emergency department visits for vertigo, nausea, vomiting, and abdominal pain. During this time, the results of blood work and computed tomography (CT), esophagogastroduodenoscopy, and colonoscopy examinations were inconclusive, and the patient was suspected to have a possible psychogenic component to her symptoms.

## 3 Methods

### 3.1 Differential Diagnosis and Investigations

In the 2 years after initial presentation, the patient was admitted to different hospitals and treated by various specialists, including gastroenterologists and rheumatologists, for her disabling nausea, vomiting, and abdominal pain. Five months after the initial presentation, a hepatobiliary iminodiacetic acid scan identified questionable gallbladder dyskinesia. As a result, laparoscopic cholecystectomy surgery was performed, but her symptoms did not improve. In months 5–8 after the initial presentation, a CT scan of her abdomen and pelvis revealed mild peripancreatic stranding; laboratory tests revealed an elevated lipase level (1500 U/L) and antinuclear antibodies, ribonucleoprotein, and Sjögren syndrome–related antigen A, but the IgG4 test result was negative. Possible autoimmune pancreatitis or systemic lupus erythematosus was suspected, and the patient received treatment with high‐dose intravenous methylprednisolone plus oral prednisone. The patient responded to steroid therapy after an initial nonresponse and was asymptomatic with an unremarkable CT of the abdomen and pelvis and lipase values within the reference range. However, in months 9–17 after the initial presentation, the patient again experienced nausea, vomiting, and abdominal pain plus hip pain. Liver biopsy results suggested chronic hepatitis with Stage 1/4 inflammatory activity and Stage 1/4 fibrosis. The patient was still considered to have autoimmune pancreatitis and restarted prednisone. Later, her hip pain was determined to be caused by avascular necrosis secondary to chronic systemic steroid use, and her gastrointestinal symptoms were suspected to be functional or psychogenic. She was referred to orthopedics for steroid‐induced avascular necrosis and prescribed a tricyclic antidepressant for functional bowel disease. In months 18–21 after the initial presentation, the patient suddenly developed new symptoms including itching in the back, numbness below the breast, left extremity weakness, urinary incontinence, and stool impaction. Her neurological symptoms were acute, progressed rapidly within a few days, and prompted MRI. Initial MRI was remarkable; her entire cervical and thoracic spinal cord was enlarged with high T2 signal throughout the central spinal cord and mild patchy enhancement noted after contrast administration. There was no abnormal leptomeningeal enhancement or epidural collections. Based on the imaging results, radiologists suggested differential diagnoses including acute disseminated encephalomyelitis, neuromyelitis optica, viral myelitis, and idiopathic transverse myelitis. However, rheumatologists were under the impression that the transverse myelitis was due to systemic lupus erythematosus. The patient started treatment with intravenous methylprednisolone and plasma exchange, followed by cyclophosphamide plus prednisone, and underwent multiple rehabilitation admissions for paraplegia. After completing 3 monthly infusions of cyclophosphamide, the patient experienced improvement in the spinal cord inflammation on MRI but still had increased T2 signal from cervical vertebra 3 to thoracic vertebra 2. Her symptoms continued with no meaningful improvement of left extremity weakness after treatment with cyclophosphamide, and only then was she referred to neurologists for consultation.

In months 22–24 after the initial presentation, neurology examinations confirmed left extremity weakness (strength 3/5; wheelchair‐bound), pain/spasm and burning sensation in legs, and urinary urgency; nausea and vomiting had resolved at the time. MRI of the brain was unremarkable, but MRI of the cervical, thoracic, and lumbar spine revealed cord expansion with high T2 signal and patchy enhancement in the entire cervical and thoracic regions (Figure 1), as well as in the lumbar region. A follow‐up MRI revealed extensive T2 lesions and significant cord atrophy. Neurologists in the consultation raised the possibility of NMOSD and obtained lumbar puncture. Lab evaluations revealed positive Sjögren syndrome–related antigen A, antinuclear antibodies for possible rheumatological diseases, and positive AQP4‐IgG. The cerebrospinal fluid examination revealed findings consistent with NMOSD, with high white (497 cells/μL) and red (68 cells/μL) blood cell counts, predominance of polymorphonuclear leukocytes (59%), elevated protein level (300.1 mg/dL), enhanced IgG synthesis (94.05 mg/24 h), and elevated IgG index (0.73) but no oligoclonal bands. The patient was initially tested for serum AQP4‐IgG antibodies by ARUP Laboratories using an enzyme‐linked immunosorbent assay (ELISA), which revealed a highly positive titer of > 160. Because of the low specificity and concern about false positivity of ELISA, the patient was tested again by the Mayo Clinic to confirm diagnosis using a cell‐based assay, which was also highly positive at a titer of 113. Cell‐based assays are generally regarded as the most sensitive approach for AQP4‐IgG detection because they preserve native antigen conformation and improve recognition of clinically relevant antibodies [1, 2, 14, 15]. Given the patient's neurological symptoms, MRI and cerebrospinal fluid findings, and positive serum AQP4‐IgG result, she was finally diagnosed with AQP4‐Ab+ NMOSD 2 years after her initial symptom presentation.

**FIGURE 1:**
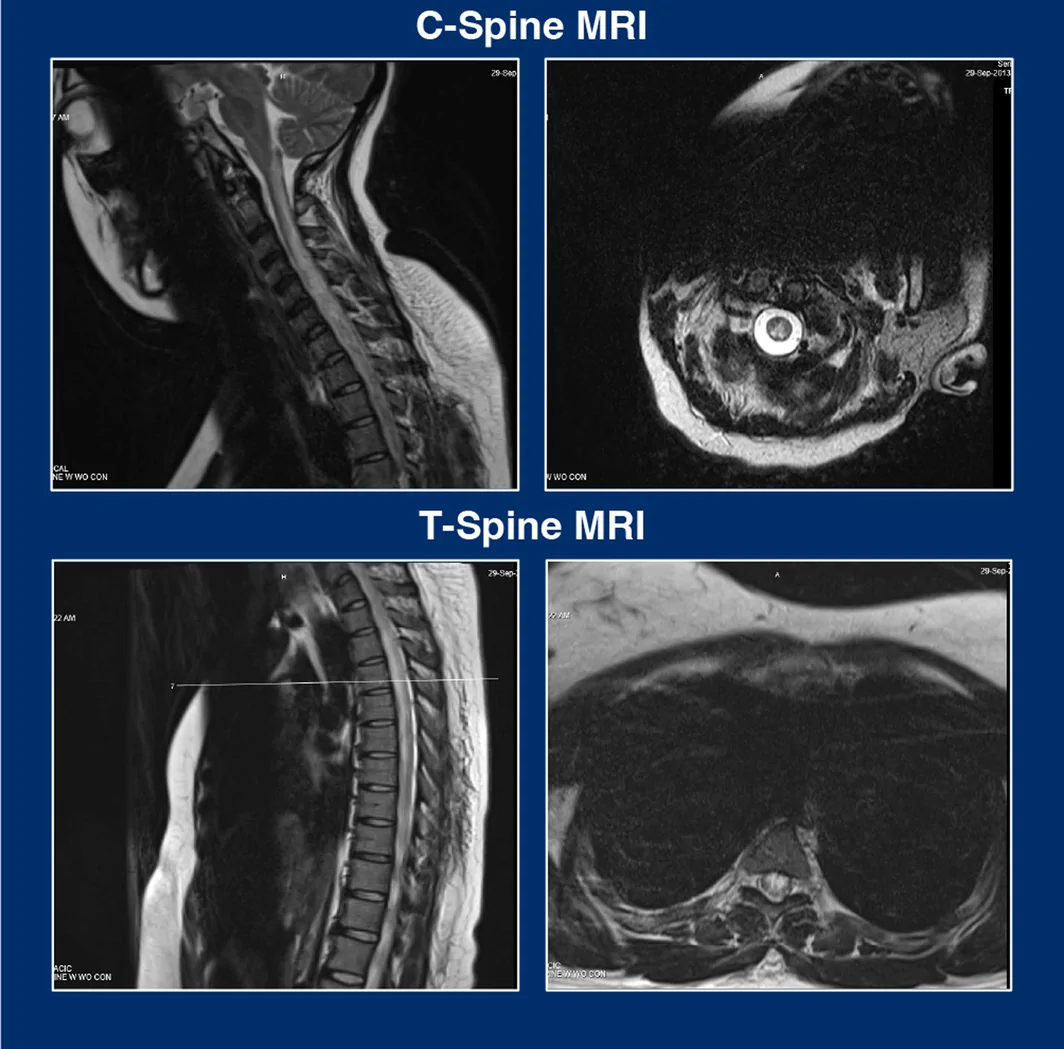
MRI of the patient's C‐spine and T‐spine. MRI of the C‐spine reveals diffuse cord expansion, abnormally increased T2 signal throughout the entire cervical region, and patchy enhancement after contrast administration. There were no mass lesions. MRI of the T‐spine shows that the entire thoracic cord was enlarged with high T2 signal throughout the central cord and mild patchy enhancement after contrast administration. There were no abnormal leptomeningeal enhancement or epidural collections and no focal mass or syrinx, and the appearance of the thoracic vertebral bodies and discs was normal. C‐spine, cervical spine; MRI, magnetic resonance imaging; T‐spine, thoracic spine.

### 3.2 Treatment

After receiving the diagnosis, the patient commenced rituximab 1000 mg on Days 1 and 15 with repeated cycles every 6 months and was treated for a total of ~4 years (Figure 2). Throughout the course of rituximab treatment, the patient was relatively stable clinically. However, occasional symptom flare‐ups prompted monitoring of the patient for B‐cell depletion at various frequencies. Although the numbers of CD19+ and CD20+ B cells were low most of the time, the patient demonstrated B‐cell repopulation throughout the 4 years of rituximab treatment (Figure 3). Given the established association between B‐cell reconstitution and increased relapse risk among patients with AQP4‐IgG–positive NMOSD treated with rituximab [16, 17], we considered this to represent suboptimal treatment durability rather than treatment failure.

**FIGURE 2:**
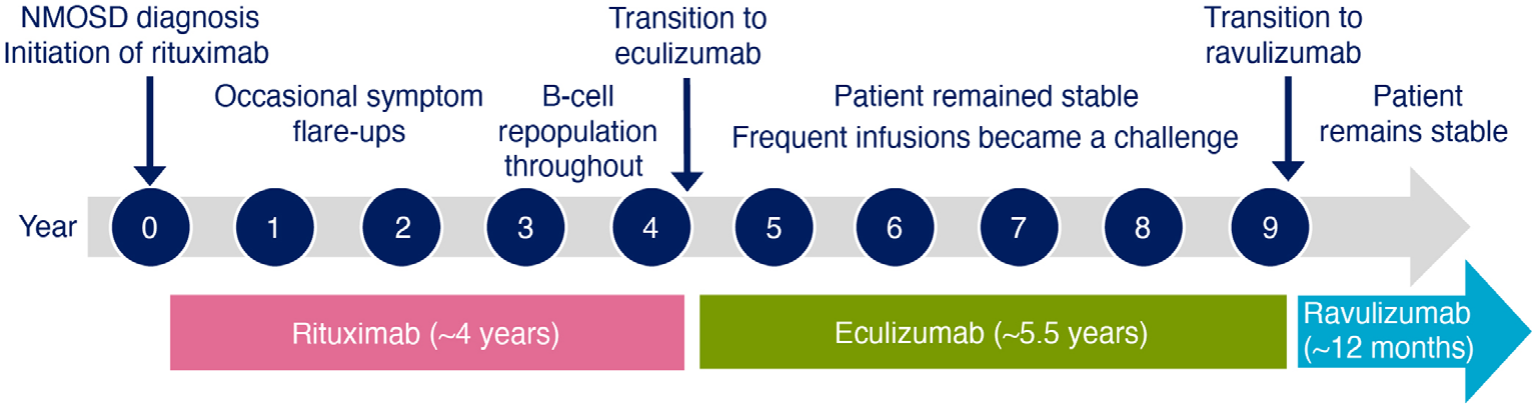
Timeline of immunotherapy administered since the patient's diagnosis of NMOSD. NMOSD, neuromyelitis optica spectrum disorders.

**FIGURE 3:**
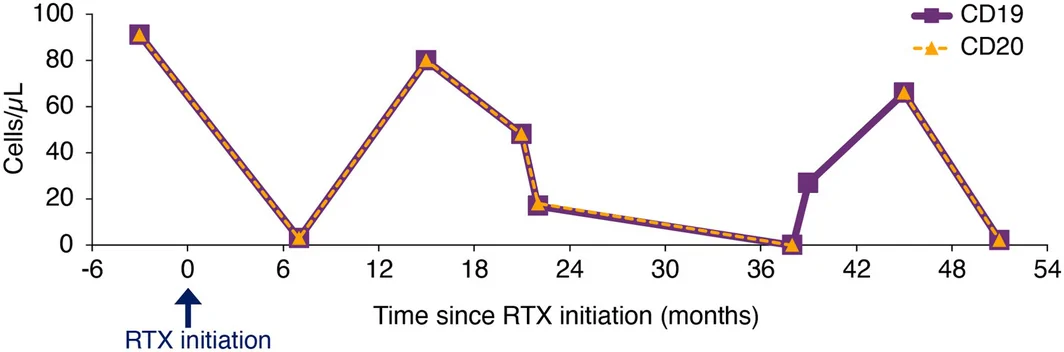
Number of B cells over time. The patient received 1000 mg of rituximab on Days 1 and 15 with repeated cycles every 6 months for ~4 years. The patient demonstrated B‐cell repopulation throughout the 4 years of rituximab treatment. RTX, rituximab.

Concerns about B‐cell repopulation occurring early and possible breakthrough disease on rituximab led to the decision to transition the patient from rituximab to eculizumab despite no new or worsening symptoms at the time (Figure 2). The lymphocyte count (952 cells/μL) was close to the reference range at treatment transition. Eculizumab treatment was initiated ~2.5 months after the last dose of rituximab and was administered intravenously at 900 mg weekly for 4 weeks, followed by a 1200 mg dose 1 week later and then 1200 mg/week every 2 weeks thereafter. Before and during the start of eculizumab treatment, the patient received 2 types of meningococcal vaccines, including the meningococcal B outer membrane vesicle (OMV) adjuvanted vaccine 3.5 months before the first dose of eculizumab and on the day of the first dose of eculizumab and the meningococcal ACWY conjugate vaccine on the day of the first dose of eculizumab and 2 months after the first dose of eculizumab. No prophylactic antibiotics were prescribed for the patient as she was deemed to be at very low risk of exposure for meningococcal infection given her isolated lifestyle.

The patient was stable on eculizumab; however, frequent infusions posed a challenge because of the loss of peripheral intravenous access, which necessitated port placement. The patient underwent hip replacement surgery for treatment of avascular necrosis secondary to chronic systemic steroid use, which was necessary owing to the delayed diagnosis of NMOSD, after ~4.5 years of eculizumab treatment. The surgery helped with ambulation, but coordinating eculizumab infusions during hospital and rehabilitation admissions required special attention. She was re‐evaluated by a physical therapist and discharged with home physical therapy to receive eculizumab in an ambulatory infusion center for another year.

After a total of 5.5 years of eculizumab therapy, the patient and her treating physician jointly decided to transition to ravulizumab for the convenience of Q8W dosing (Figure 2). The logistical difficulties with Q2W eculizumab infusions, poor intravenous access, and the hardship of coordinating frequent infusions with rehabilitation appointments were the reasons for the therapeutic transition, not lack of efficacy. The patient underwent MRI of the cervical and thoracic spine approximately 1 year before the last dose of eculizumab, and the results remained stable. The patient also was clinically stable with no new relapses and no new or worsening comorbidities or symptoms at the time of treatment switch. Her lymphocyte count (1378 cells/μL) was within the reference range at ravulizumab treatment initiation. Ravulizumab loading and maintenance doses were based on the patient's weight. The loading dose was initiated 2 weeks after the last dose of eculizumab, and the maintenance dose was initiated 2 weeks after the ravulizumab loading dose and maintained every 8 weeks. Because of the long period of therapy with C5 inhibitors, the patient received the meningococcal B OMV adjuvanted vaccine 6 days prior to and on the same day of the last dose of eculizumab (2 weeks before the first dose of ravulizumab). The meningococcal ACWY conjugate vaccine was given 6 months after the first dose of ravulizumab. As with the initial meningococcal vaccines, no antibiotics were used. The patient had insurance coverage for ravulizumab and continued to receive her infusions at the ambulatory infusion center. Transportation challenges were more manageable after the transition to Q8W ravulizumab dosing. To date, the patient has been on ravulizumab for 12 months, and the treatment is ongoing. The patient is stable with no disease relapse and no treatment‐related adverse events or complications.

## 4 Conclusion

In this case report, a patient with AQP4‐Ab+ NMOSD was not diagnosed for 2 years and had continuing relapses while on various treatments. Rituximab was effective in treating the patient, but early B‐cell repopulation necessitated transition to C5 inhibitors. Both eculizumab and ravulizumab successfully maintained disease remission, and the longer dosing interval of ravulizumab provided further logistical benefit for the patient. The patient is pleased with the effectiveness and convenience of ravulizumab treatment. The ability to have an infusion every 8 weeks has significantly reduced logistical challenges the patient faced with her former treatment that required more frequent dosing. The patient understands the importance of staying up to date with meningococcal vaccination and remains vigilant for signs of infection. Overall, the patient is satisfied with her treatment journey and values the stability ravulizumab has provided in managing her NMOSD.

## 5 Discussion

Targeted immunotherapies have transformed the therapeutic landscape for patients with AQP4‐Ab+ NMOSD [8]. Although rituximab is frequently used off‐label for NMOSD, inadequate response and/or breakthrough attacks are a concern for some patients [8, 9, 10, 11]. Eculizumab and ravulizumab are US Food and Drug Administration–approved C5 inhibitors indicated for treating AQP4‐Ab+ NMOSD [18, 19], with efficacy in reducing relapse risk proven in pivotal Phase 3 trials [12, 13]. There is a sparsity of real‐world data on the transition from B‐cell depletion therapy to C5 inhibition therapy. This case report of a patient who switched from rituximab to eculizumab and then to ravulizumab and maintained remission without relapse provides insight into the efficacy and real‐world application of therapy transition to C5 inhibitors.

Patients who had received rituximab within 3 months before screening were excluded from the pivotal Phase 3 clinical trials of eculizumab and ravulizumab, precluding evaluation of the effects of switching treatments [12, 13]. The therapeutic response to C5 inhibition observed in this patient when she had early B‐cell repopulation and possible breakthrough on rituximab supports the use of targeted complement inhibition as relapse‐preventive therapy after rituximab.

Ravulizumab, a long‐lasting C5 inhibitor, has the potential to reduce treatment burden for patients [13]. Alleviation of transportation challenges when this patient switched from Q2W eculizumab to Q8W ravulizumab underscores the importance of considering the practical implications of therapies when treating patients. Switching from eculizumab to ravulizumab was based on patient preference for a more convenient dosing schedule. The patient‐centric care in treatment decision‐making or choices helps patients maintain uninterrupted access to effective therapies.

This case report also highlights real‐world scenarios and logistics of transitioning from B‐cell depletion therapy to C5 inhibition therapy. Meningococcal B and ACWY vaccinations were started before and during transition from rituximab to eculizumab treatment because of the increased risk of infections by *Neisseria meningitidis* associated with C5 inhibition. For transition from eculizumab to ravulizumab, the loading dose of ravulizumab was simply administered at the time of the next scheduled eculizumab dose (i.e., 2 weeks). As the patient was on eculizumab for 5.5 years and then switched to ravulizumab, meningococcal booster shots were administered during eculizumab treatment and before and during ravulizumab treatment. Prophylactic antibiotics were not prescribed for this patient as she was deemed to be at very low risk of exposure for meningococcal infection. Administration of prophylactic antibiotics should be considered for patients who are active and spend any time in populated areas, who are disabled and live in nursing facilities, and who are not up to date with their meningococcal vaccines and require urgent eculizumab therapy to reduce the risk of meningococcal infection.

Limited knowledge and awareness of NMOSD, variability in clinical presentations and disease courses, and shared clinical features with multiple sclerosis or other autoimmune neurologic diseases are some factors that can lead to missed diagnosis of NMOSD [20]. In this case study, the patient's initial presentation of vertigo, nausea, vomiting, and abdominal pain, coupled with unfamiliarity with NMOSD among gastroenterologists and rheumatologists, may have resulted in the delay in acquiring MRI, neurology consultation, and serum AQP4‐IgG testing, which consequently contributed to her delayed diagnosis. Area postrema clinical syndrome, consisting of intractable hiccups or nausea and vomiting, is one of the six criteria for NMOSD diagnosis [1]. Differential diagnosis of NMOSD may be difficult as there are shared clinical features between NMOSD and other common autoimmune diseases [20], such as multiple sclerosis (prevalence: 35.9 per 100,000 persons; incidence: 2.1 per 100,000 persons/year) [21], systemic lupus erythematosus (prevalence: 43.7 per 100,000 persons; incidence: 5.1 per 100,000 persons/year) [22], Sjögren's syndrome (prevalence: 0.01%–0.05%; incidence: 1–10 per 100,000 persons/year) [23], and antiphospholipid syndrome (prevalence: 40–50 per 100,000 persons; incidence: 1–2 per 100,000 persons/year) [24]. Strategies for overcoming challenges associated with the diagnosis of NMOSD are needed to help physicians efficiently diagnose patients, thereby enabling delivery of effective therapies. This case highlights the therapeutic efficacy of using C5 inhibition therapy after B‐cell depletion therapy, as well as the urgency to diagnose and treat NMOSD early to prevent and reduce relapses and disability.

## Author Contributions

**Aram Zabeti:** conceptualization, data curation, investigation, project administration, writing – original draft, writing – review and editing.

## Funding

Medical writing and editorial support of this manuscript were funded by Alexion, AstraZeneca Rare Disease.

## Consent

The patient provided written informed consent to participate in this study. Written informed consent was also obtained from the patient for the publication of any potentially identifiable images or data included in this article.

## Conflicts of Interest

Aram Zabeti: Research funding from NIH, PCORI, Alexion, Genentech, Chugai, Medimmune, and Biogen; honoraria for Speaker Bureau or Advisory Board from Alexion/AstraZeneca, Biogen, Celgene/Bristol Myers Squibb, Genentech‐Roche, Novartis, EMD‐Serono, Teva Pharma, Sanofi‐Genzyme, Viela Bio/Horizon/Amgen, and TG therapeutic.

## Data Availability

The author declares that all supporting data are available within the article. Further inquiries can be directed to the author.
